# Neuronal Orphan G-Protein Coupled Receptor Proteins Mediate Plasmalogens-Induced Activation of ERK and Akt Signaling

**DOI:** 10.1371/journal.pone.0150846

**Published:** 2016-03-02

**Authors:** Md. Shamim Hossain, Kurumi Mineno, Toshihiko Katafuchi

**Affiliations:** Department of Integrative Physiology, Graduate School of Medical Sciences, Kyushu University, Fukuoka, Japan; University of Louisville, UNITED STATES

## Abstract

The special glycerophospholipids plasmalogens (Pls) are enriched in the brain and reported to prevent neuronal cell death by enhancing phosphorylation of Akt and ERK signaling in neuronal cells. Though the activation of Akt and ERK was found to be necessary for the neuronal cells survival, it was not known how Pls enhanced cellular signaling. To answer this question, we searched for neuronal specific orphan GPCR (G-protein coupled receptor) proteins, since these proteins were believed to play a role in cellular signal transduction through the lipid rafts, where both Pls and some GPCRs were found to be enriched. In the present study, pan GPCR inhibitor significantly reduced Pls-induced ERK signaling in neuronal cells, suggesting that Pls could activate GPCRs to induce signaling. We then checked mRNA expression of 19 orphan GPCRs and 10 of them were found to be highly expressed in neuronal cells. The knockdown of these 10 neuronal specific GPCRs by short hairpin (sh)-RNA lentiviral particles revealed that the Pls-mediated phosphorylation of ERK was inhibited in *GPR1*, *GPR19*, *GPR21*, *GPR27* and *GPR61* knockdown cells. We further found that the overexpression of these GPCRs enhanced Pls-mediated phosphorylation of ERK and Akt in cells. Most interestingly, the GPCRs-mediated cellular signaling was reduced significantly when the endogenous Pls were reduced. Our cumulative data, for the first time, suggest a possible mechanism for Pls-induced cellular signaling in the nervous system.

## Introduction

Plasmalogens (Pls), which are glycerophospholipids characterized by the presence of vinyl ether linkage at the *sn-1* position, are enriched in the central nervous system [1,2]. Pls are not only the structural membrane components and reservoirs for second messengers, but also reported to play a role in the membrane fusion, ion transport and cholesterol efflux [3]. In addition, since the vinyl ether bond at the *sn*-1 makes Pls more susceptible to oxidative stress than corresponding ester bonded glycerophospholipids, Pls act as antioxidants and protect cells from oxidative stress [4]. Based on the evidence that Pls were reduced in the Alzheimer’s patients brain samples [4–6], we previously hypothesized that they might have a neuroprotective function and in fact found their role as anti-apoptotic agents [7]. Pls were found to induce phosphorylation of ERK and Akt kinases resulting in the inhibition of apoptotic cleavage of caspase-3 to inhibit neuronal cell death [7]. The independent research group also reported that Pls had the capability to induce phosphorylation of Akt resulting in the myelination of axons by Schwan cells in the peripheral nervous system [8]. It is, therefore, reasonable that Pls-mediated induction of cellular signaling can play a role in the nervous system [3,4]. However, the precise mechanism for the Pls-induced cellular signaling is mostly unknown.

We previously found that Pls induced phosphorylation of Akt and ERK in neuronal cells but not in astrocytes [7]. To identify the possible target proteins involved in the Pls-induced cellular signaling in brain cells, we have screened several orphan G-protein coupled receptors (GPCRs). It is a common understanding that many GPCRs function through the membrane lipid rafts which are known to be enriched with the Pls [4,9–11]. Lipid rafts are subdomains in the cell membrane containing cholesterol, glycosphingolipids and other cellular components at high concentrations and forming a platform for many membrane proteins to transduce their signaling [10,12]. It is also known that a receptor for one of glycerophospholipids, platelet-activating factor (PAF) is a GPCR [13,14]. To this hypothesis, we found interesting evidence that the Pls-mediated phosphorylation of Akt and ERK in neuronal cells was inhibited by pre-treatment with a G-protein inhibitor, suggesting that GPCRs might be possible mediators of Pls-signaling cascade. We then screened several orphan GPCRs based on their high abundance in the nervous system and identified 5 neuronal specific orphan GPCRs that might play a role in enhancing Pls-signaling in neuronal cells.

## Materials and Methods

All experimental procedures involving the usage of animals were approved by the Ethics Committee on Animal Experiments at Kyushu University, Japan. We have strictly followed the guidelines “Principles for the Care and Use of Animals” described by the Physiological Society of Japan. All efforts were made to minimize animal’s suffering and the number of animals used for study.

### Preparation of Pls

Highly pure Pls were prepared by extracting from chicken skin as reported previously [15] and kindly donated by Central Research Institute, Marudai Food Co. Ltd. (Osaka). Pls consisted of 96.5% ethanolamine Pls, 2.5% choline Pls, 0.5% sphingomyelin and 0.5% other phospholipids. Pls were dissolved in 99.5% ethanol to the stock concentration of 10 mg/ml and diluted to the desired concentration (500 ng/ml) immediately before use. Vehicle (ethanol at the same concentration without Pls) was used in the control groups.

### Cell cultures

The human embryonic kidney derived 293T (Hek293T) cells, mouse neuroblastoma derived cells, (Neuro 2A, or N2A), astrocyte cell lines (A1) and microglial cell lines (MG6) were purchased from Health Science Research Resources Bank, RIKEN, Japan. Cells were maintained in Dulbecco’s modified Eagle’s medium (DMEM) containing 10% heat-inactivated fetal bovine serum (FBS) (Invitrogen, Carlsbad, CA, USA), 50 μg/ml penicillin, 50 μg/ml streptomycin (Invitrogen) and glucose at 37°C in 5% CO_2_ as described before [7].

Primary hippocampal neurons were prepared from E-18 embryo of mice. After dissection of anesthetized pregnant mice, meninges of embryo were removed carefully. The hippocampus were cleared with the surrounding cortex and dissolved in trypsin solution containing PBS, bovine serum albumin (BSA), and glucose at 37°C for 15 minutes. FBS was used to neutralize the trypsin activity. The hippocampus were then dissociated in neurobasal medium (GIBCO) supplemented with B27 (GIBCO) by appropriate pipetting using different pour sized Pasteur pipette. The dissociated neurons were then cultured on poly-D-lysine coated glass cover slips (30,000 cells/15 mm coverslip) with neurobasal medium in 5% CO_2_ humidified incubator. On DIV (Disk In Vitro) 3, 90% of cultured medium was replaced with B27 free neurobasal medium. Cytosine arabinoside (Ara-C) purchased from Sigma was added on DIV 3 primary neurons at a concentration of 1 μM to inhibit microglial proliferation. More than 95% pure primary hippocampal pyramidal neuronal cells (on disk *in vitro* 21) were used as primary neurons [7]. Primary microglia (>90% pure) and astrocytes (>85% pure) were collected according to our previous report [16] from the hippocampal tissue of the new born mice.

### Real-time PCR analyses

Total RNA was extracted from the cells by TRIZOL reagents (Life Technologies) following standard protocols. cDNAs were prepared from the purified total RNA using ReverTra Ace qPCR RT Kit (Toyobo, Japan). Real-time PCR reaction was carried out by SYBR Premix Ex Taq (Takara, Japan) following the manufacturer’s protocol. The real-time quantifications were carried on a 7500 Real Time PCR System (Applied Biosystems). The specific primers used to amplify the each mouse gene from the cDNA were as follows: *GPR1*, forward 5΄-TCTTCCAGTCTCCCAGCTTC-3΄ and reverse 5΄-TAAGCTGCGCACCCTTTCTA-3΄; *GPR15*, forward 5΄-ACAAGCCCAGATCCTCCTTT-3΄ and reverse 5΄-CCCACCACTCCAGTCAAGAA-3΄; *GPR19*, forward 5΄-ACTTCCTGCTTGGTCACAGG-3΄ and reverse 5΄-TCGTGATAGGGCAAAAATCC-3΄; *GPR20*, forward 5΄-TGGGTCCACAGACCTAGAGC-3΄ and reverse 5΄-GCATGTCAGTGGTCAGTGCT-3΄; *GPR21*, forward 5΄-TGGCTTTTGTTTGGATTTCA-3΄ and reverse 5΄-GGGCAGAGGGAGGAAGATTA-3΄; *GPR25*, forward 5΄-CTGCTTGGAACCTGCCTTAC-3΄ and reverse 5΄-TCAGTAGCCACACCACGAAG-3΄; GPR26, forward 5΄-CACGTACCTCAAGGTGCTCA-3΄ and reverse 5΄-CACACTGGGGTGTATGTCCA-3΄; *GPR27*, forward 5΄-CTTCCTGGCCGCACTCTT-3΄ and reverse 5΄-GGCGTAGAAGCGGTGGTG-3΄; *GPR50*, forward 5΄-CCGAAATTCTGGCAACATCT-3΄ and reverse 5΄-CCCAACTGACATGGCATACA-3΄; GPR52, forward 5΄-ATACGCTGACCTCCTCGTTG-3΄ and reverse 5΄-ATCCAAACACCTGGCAAGTC-3΄; GPR61, forward 5΄-AGGCATCAGCTGAGAAGAGC-3΄ and reverse 5΄-TCTGCAACTCTTCCGGACTC-3΄; *GPR62*, forward 5΄-CTAGCAAACCCCAGTGAAGC-3΄ and reverse 5΄-CTACGAGAACCGCCAGGATA-3΄; *GPR82*, forward 5΄-AGCAGACCACTGTGACAACG-3΄ and reverse 5΄-TGGTTGAATGCATGTCGAGT-3΄; GPR101, forward 5΄-AGCCTTCAGCAGCAACAGAT-3΄ and reverse 5΄-GTGAAGTCAGACAGGCACGA-3΄; *GPR135*, forward 5΄-GCTCCTGCTCATCTTCTTGC-3΄ and reverse 5΄-AGGATGAAGGCGTTTGTGAC-3΄; GPR142, forward 5΄-CCCTGTGTGGCTGGTATCAT-3΄ and reverse 5΄-TAGGAGGGTTTCCTGGTCCT-3΄; *GPR150*, forward 5΄-TCTGAGCAAGGAGCCTCTGT-3΄ and reverse 5΄-CAGGATGATCCCCAAGAAGA-3΄; *GPR162*, forward 5΄-CTCGTCGGGAGTGCGTCT-3΄ and reverse 5΄-GTGGGTGTCTTGGTGCACAG-3΄; and the internal control *GAPDH*, forward 5΄-CAATGTGTCCGTCGTGGATCT-3΄ and reverse 5΄-GTCCTCAGTGTAGCCCAAGATG-3΄. We normalized the expression of target genes with the endogenous GAPDH expression by the technique of delta-delta Ct values.

### Cloning of the sh-RNA lentiviruses and the preparation of lentiviruses

The sh-RNA sequences were cloned into the pLL3.7 lentiviral vector following the protocol provided in the Addgene website (Plasmid No 11795) [17]. The target sequences of mice genome were as follows: sh-*GPR1* (5΄-GGAGGTCCTACCTTATAC-3΄), sh-*GPR19* (5΄-ATACCATCGTCTACCCGC-3΄), sh-*GPR21* (5΄-CCAGCGAGGACTAAAGGG-3΄), sh-*GPR27* (5΄-ACGCACCTCGTCTACCTC-3΄), sh-*GPR61* (5΄-AGAACGTGGTAACCTGGA-3΄), sh-*GPR62* (5΄-GCCTGAACGTTCAGATGG-3΄), sh-*GPR135* (5΄-GGTGATCGTGAAGCATCG-3΄), sh-*GPR142* (5΄-CCCAAGATACGATGGTGT-3΄), sh-*GPR150* (5΄-GAACAACCTATACAAACA-3΄), sh-*GPR162* (5΄-GGCACCTGTGACGACTAC-3΄) and sh-*GNPAT* (5΄-GGCTCAATCGGAACACGT-3’. To produce viruses, we transfected the Hek-293T cells with the cloned pLL3.7 vectors along with the packaging vectors pMD2.G (Addgene plasmid 12259), pRSV-Rev (Addgene plasmid 12253), and pMDLg/pRRE (Addgene plasmid 12251) by using the transfection reagent FuGENE-HD (Clontech). Seventy two hours after the transfection, the cells supernatant was cleared off the cell debris and centrifuged at 24,000 rpm for 3 h at 4°C and the viral pellet was dissolved in PBS buffer containing 1% BSA. After checking the viral titer in N2A cells, 2×10^5^ TDU (transduction units) were infected to the target N2A cells cultured at the number of 2×10^5^ cells/well in 6 well dishes.

### Cloning of G-protein coupled receptors and overexpression in the cells

The mouse mRNA sequences of the gene *GPR1*, *GPR19*, *GPR21*, *GPR61* were cloned by the PCR from the cDNA derived from mouse embryo of E16. The high fidelity polymerase enzyme (LA-tag, TAKARA) was used to clone the gene sequences and sub cloned into the T-vector (pGEM-T Easy, Promega) followed by the confirmation of the sequences. The following primer sets were used for sub-cloning *GPR1* (forward: 5΄-GTTTGAGGCTAGAAAGGGTGCGCA-3΄ and reverse: 5΄-CAGAGGGCAGCCAGAAAGATACATG-3΄); *GPR19* (forward: 5’-GGAGATATGAATGTGTTCCGA-3’ and reverse: 5’- AATCTCTGGCACATAACAGTGA-3’); *GPR21* (forward: 5’-CAGTATACCCACCACAGCAGCAAC-3’; and reverse: 5’- CTATGAAGGTTATCTGGATAGTCTG-3’); and *GPR61* (forward: 5’-AGTGATTAGAGCCTGCCTTACAGG-3’ and reverse: 5’- TGCCACATGTCCATCCTGTCGATTC—3’). After confirming the sequences of the cloned genes, the ORF (open reading frame) sequences were cloned by infusion HD (Takara, Japan) techniques by following the standard protocol into the expression plasmid c-Flag pCDNA3 (Addgene vector no 20011). The following primer sets were used for clone the ORF sequences into the c-Flag pCDNA3 plasmid vectors: *GPR1* (forward: 5’ CTTATCTAGACTCGA**GCCACC**ATGGAAGTCTCAAAGGAAATGT-3’ and reverse: 5΄-ATCCGGTACCCTCGAGCTGGGCAGTTTCTAGGAGAG-3’); *PR19* (forward: 5’-CTTATCTAGACTCGA**GCCACC**ATGGATAACGACCAGCCGCCTG-3’ and reverse: 5΄-ATCCGGTACCCTCGAGGACAAAAGTGTTTGGAGGGT-3’); *GPR21* (forward: 5’-CTTATCTAGACTCGA**GCCACC**ATGAACTCCACCTGGGATGGTA-3’ and reverse: 5΄-ATCCGGTACCCTCGAGGATATGAGATCCATTAGG-3’); and *GPR61* (forward: 5’-CTTATCTAGACTCGA**GCCACC**ATGGAGTCCTCACCCATCCCC-3’ and reverse: 5΄-ATCCGGTACCCTCGAGTGACTCCAGCCTTGGTGAGG-3’). The underlined sequences were designed for recombination into the EcoR1 restriction enzyme digested c-Flag pCDNA3 vector. The Kozak sequences, GCCACC (bold character), were added before the first codon ATG of each genes ORF sequences aimed to enhance the protein expression. The cloned vectors were then transfected into the Hek293T and N2A cells by FuGENE-HD (Promega) and the expression was confirmed by western-blotting assays using the flag monoclonal antibody.

### Western blot analysis

Western blotting assays were performed by the protocol described in our previous report [7]. The following antibodies are used for immune blotting assays: monoclonal anti-Flag (M185-3L, MBL), Akt (610860, BD Biosciences), β-Actin (sc-4778, Santa Cruz) and the cell signaling antibodies (p-Akt, ERK, and p-ERK). GNPAT antibody was purchased from Abcam (ab75060).

### Statistical analysis

To analyze the significant differences between two groups, Student’s t-test was used. For the multiple comparisons, we performed one way ANOVA followed by Bonferroni’s test except for the comparison to a single control group, in which we used Dunnett’s post hoc test. *P* values less than 0.05 were considered as statistically significant.

## Results

### G-protein Inhibitor reduces Pls-mediated phosphorylation of Akt and ERK in the neuronal cells

**Fig 1:**
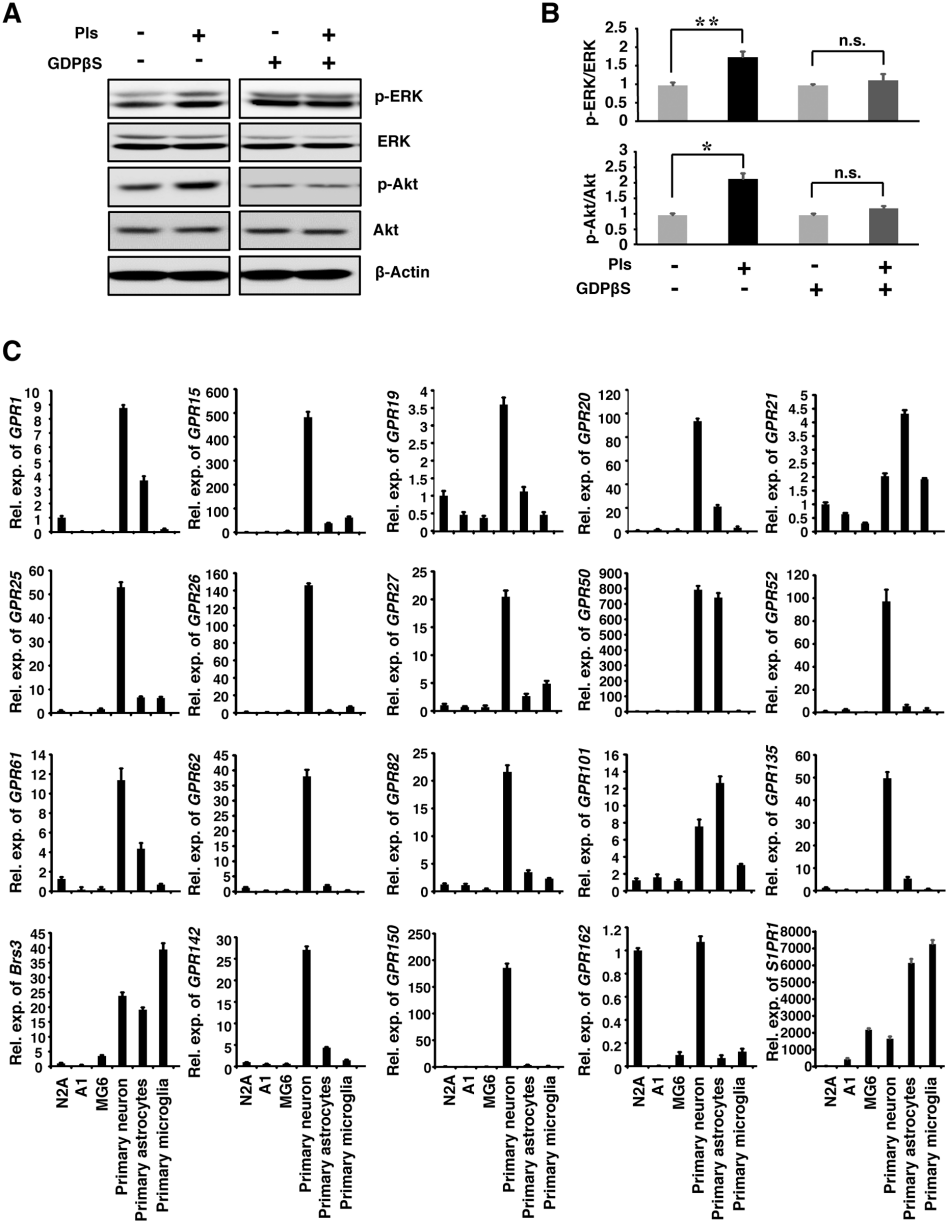
G-protein Inhibitor reduces Pls-mediated phosphorylation of Akt and ERK in the neuronal cells. (A) Pls-mediated phosphorylation of ERK and Akt was cancelled by treatments with the G-protein inhibitor. The neuronal cells (N2A) were cultured in low serum (2% FBS) containing medium for 20 hr and then treated with the G-protein inhibitor GDPβS at 200 μM concentration for 4 hours followed by Pls (500 ng/ml) treatments for 20 minutes. Cell extracts were then subjected to western blotting assays. (B) The quantification data of panel A showed that Pls treatment failed to increase phosphorylation of ERK and Akt in the GDPβS pretreated cells (One way ANOVA followed by Bonferoni’s test, n = 5, *, *P*<0.05 and **, *P*<0.01. n.s. stands for not significant). Image-J software was used to quantify the signals. (C) Real-time PCR data shows the orphan GPCRs expression in the primary neurons, astrocytes, microglia and also in the cell lines of N2A (neuronal derived cells), A1 (astrocytes) and MG6 (microglia). The data represent mean ± S.E.M (standard error of mean) (n = 3).

To see whether the Pls-mediated signaling is dependent on the GPCRs, we employed a general G-protein inhibitor GDPβS. GDPβS is known to effectively inhibit G-protein signaling when it is added extracellularly after dissolving with DMSO [18]. We treated the neuronal cells with GDPβS in DMSO and found that the extracellular addition of Pls (500 ng/ml) failed to induce phosphorylation of ERK and Akt (Fig 1A and 1B). We then looked for possible neuronal specific GPCR proteins that could participate in the signal transduction by the Pls. To screen for the possible GPCRs, we focused on orphan GPCRs that were enriched in the central nervous system. Real-time PCR data showed the mRNA expression of 19 orphan receptors among neuronal, astrocytes and microglial cells (Fig 1C). Notably, a total of 10 GPCRs (*GPR1*, *GPR19*, *GPR21*, *GPR27*, *GPR61*, *GPR62*, *GPR135*, *GPR142*, *GPR150*, and *GPR162*) were found to be highly expressed among the neuronal cells and also in the primary neuronal culture. We then hypothesized that these GPCRs might function as the mediator of the Pls-induced signaling.

### Knockdown of GPCRs inhibits Pls-mediated activation of ERK

**Fig 2:**
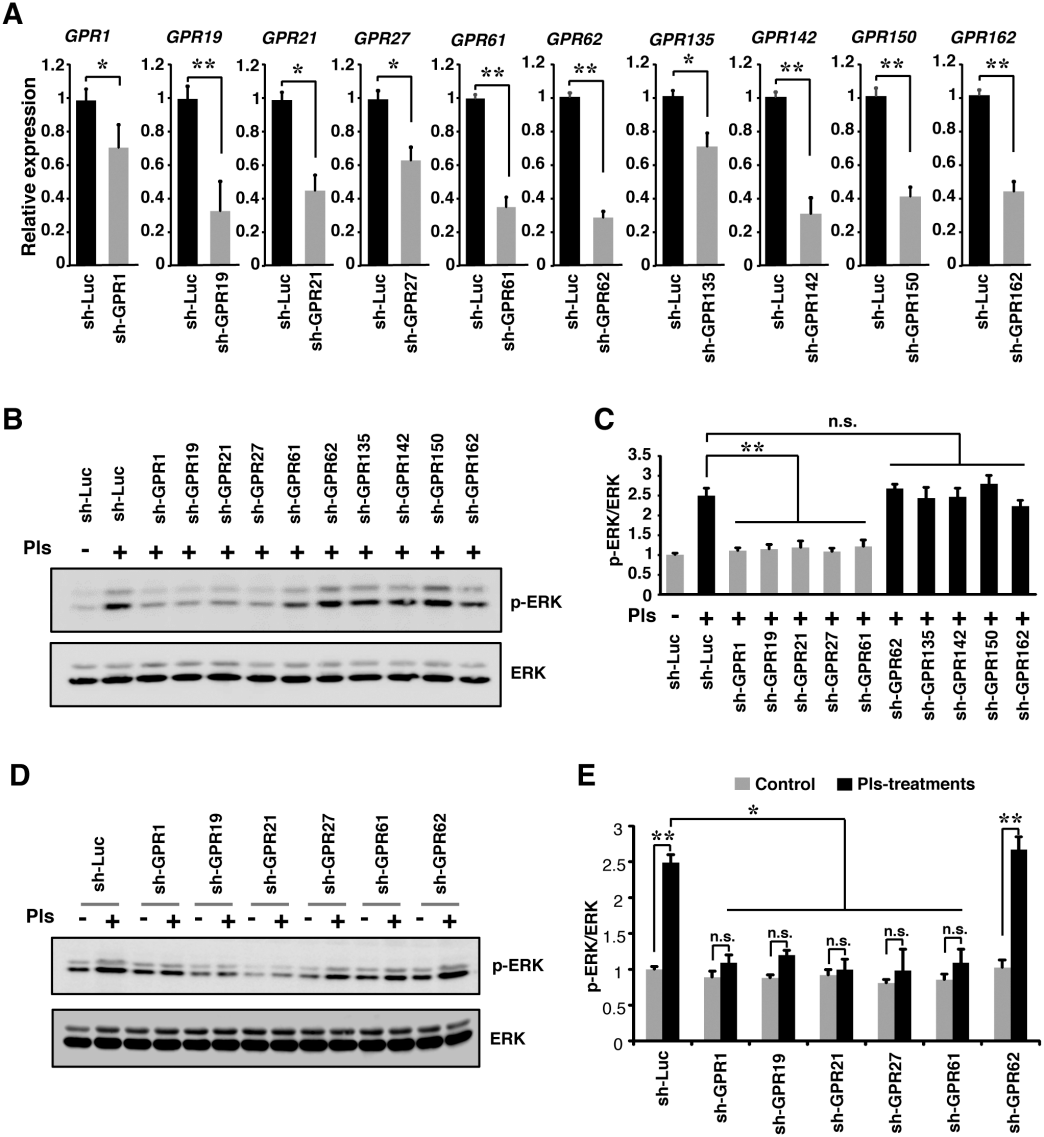
Knockdown of GPCRs inhibits Pls-mediated activation of ERK. (A) Total RNAs extracted from the sh-RNA containing lentivirus transfected N2A cells were subjected to the real-time PCR analysis. The relative expression of the target GPCRs was reduced by their respective sh-RNA (Student’s t-test, n = 5, *, *P*<0.05 and **, *P*<0.01). (B) Western-blotting assays showed the reduction of Pls-mediated phosphorylation of ERK in the *GPR1*, *GPR19*, *GPR21*, *GPR27* and *GPR61* knockdown cells. N2A cells were infected by the sh-RNAs lentiviruses followed by the treatments with 2% FBS containing medium for 24 hours. Pls (500 ng/ml) were added 20 minutes before the cells were collected for western blotting assays. The data represent three independent experiments (n = 3). (C) The quantification data of panel B showed a significant reduction in the expression of phosphorylated ERK in the *GPR1*, *GPR19*, *GPR21*, *GPR27* and *GPR61* knockdown cells treated with the Pls. The data represent mean ± S.E.M and the n.s. indicates not significant (One way ANOVA followed by Dunnett’s post hoc test by considering the sh-Luc with Pls group as reference. n = 5, *, *P*<0.05 and **, *P*<0.01). (D) Western-blotting assays showed the difference in the p-ERK expression between Pls-treated and non-treated groups of *GPR1*, *GPR19*, *GPR21*, *GPR27*, *GPR61* and *GPR62* knockdown cells. The data represents 3 independent experiments. (E) The quantification data of panel D showed no effect of Pls in the sh-*GPR1*, sh-*GPR19*, sh-*GPR21*, sh-*GPR27* and sh-*GPR61*, but not sh-Luc and sh-*GPR62* groups. The data represent mean ± S.E.M and the n.s. indicates not significant (Student`s t-test; n = 3, **, *P* <0.01). Furthermore, the reduction of p-ERK expression in the Pls treatment groups of *GPR1*, *GPR19*, *GPR21*, *GPR27* and *GPR61* knockdown cells was significant compared with the control sh-Luc group. (Dunnett’s test. n = 3, *, *P* < 0.05).

To identify the possible GPCR(s) involved in the Pls-signaling from the selected candidates, we have approached a knock down assays using the sh-RNA lentiviruses targeting the individual GPCRs in the neuronal cells. We cloned selective sh-RNAs deliverable plasmid vectors and the knockdown efficiency of the lentiviruses was confirmed by infection in the neuronal cells followed by real-time PCR analysis. A significant reduction of mRNA expression of the targeted GPCRs was confirmed in the lentivirus transfected cells (Fig 2A). We then infected the N2A cells by the sh-RNAs targeting the GPCRs and the control Luciferase and treated with Pls. Western blotting assays showed that the extracellular addition of Pls did not increase the phosphorylated ERK in the neuronal cells infected with the sh-*GPR1*, sh-*GPR19*, sh-*GPR21*, sh-*GPR27* and sh-*GPR61* (Fig 2B and 2C). Knockdown of other 5 GPCRs by the lentiviruses (sh-*GPR62*, sh-*GPR135*, sh-*GPR142*, sh-*GPR150*, and sh-*GPR162*) had same effect as that of the control sh-Luciferase (sh-Luc.) infected cells, suggesting that only the 5 orphan GPCRs among the selected candidates were involved in the Pls-induced signaling in the neuronal cells. In addition, we also compared the induction of p-ERK expression between Pls-treatment and non-treatment in the 5 selected GPCRs (*GPR1*, *GPR19*, *GPR21*, *GPR27* and *GPR61*) and *GPR62* knockdown N2A cells. We found that the treatment with Pls had no effect in the 5 selected groups, while control sh-*Luc* and *GPR62* knockdown groups showed significant effects of Pls-treatment (Fig 2D and 2E). These cumulative data suggest that the GPR1, GPR19, GPR21, GPR27 and GPR61 transduce Pls-mediated signaling.

### Overexpression of *GPR1*, *GPR19*, *GPR21* and *GPR61* enhance phosphorylation of ERK and Akt in the cells

**Fig 3:**
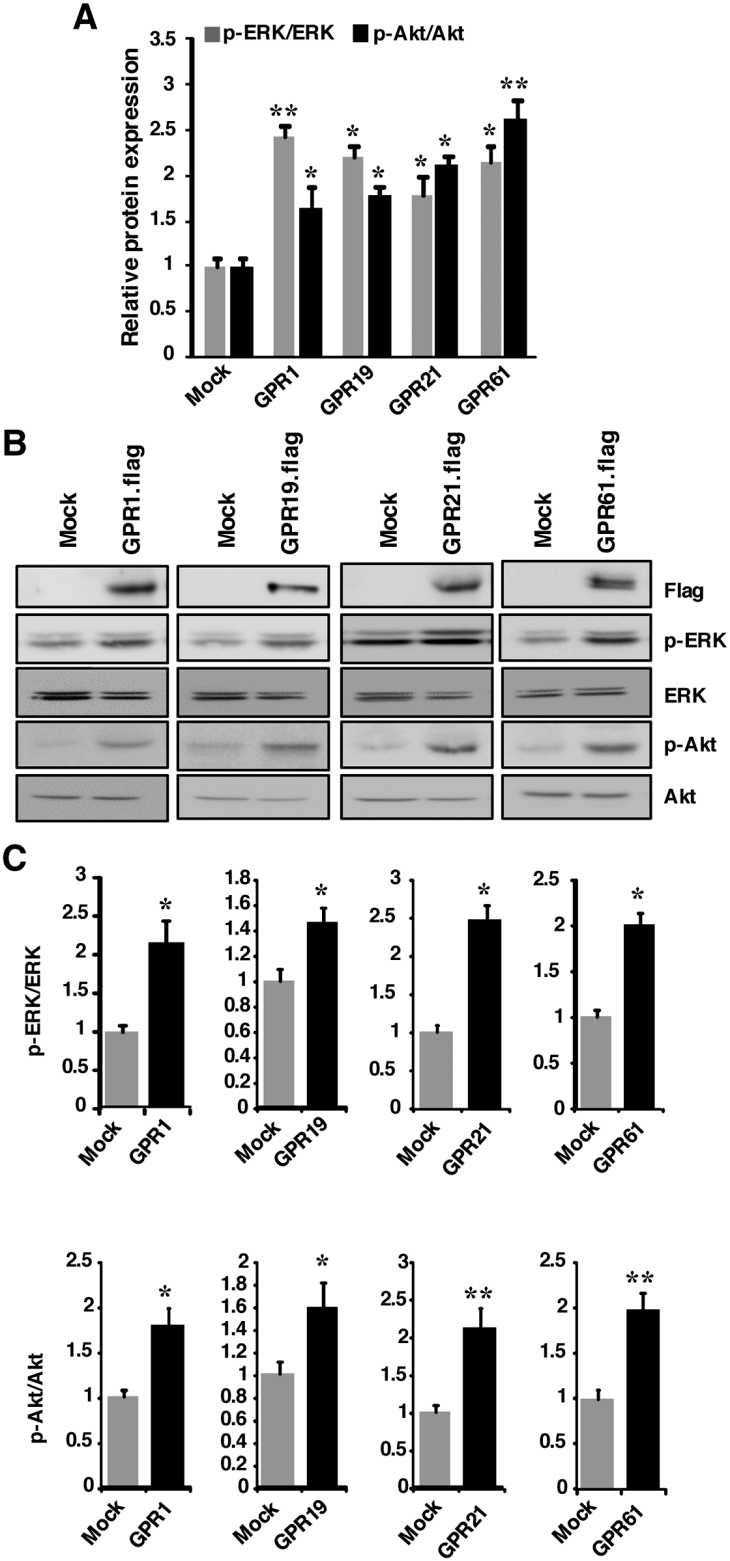
Overexpression of GPCRs enhances phosphorylation of ERK and Akt in the cells. (A) Hek293-T cells cultured in 2% FBS containing medium were transfected by the flag tagged *GPR1*, *GPR19*, *GPR21* and *GPR61* expression plasmids along with the control flag tagged empty plasmids (mock group) for 48 hours followed by western blotting assays. Overexpression of the proteins was confirmed by the immunoblotting with anti-Flag antibody. Quantification of p-ERK, p-Akt and the total proteins of ERK and Akt showed the significant increases in the phosphorylation of ERK and Akt by the overexpression of GPCRs compared with mock empty flag tagged plasmid transfected group (Dunnett’s test, n = 5, *, *P*<0.05 and **, *P*<0.01). The data represents three independent experiments. (B) Similar to that of the Hek-293T cells of panel A, N2A cells were transfected by the GPCR expressing plasmids along with the control mock empty flag tagged plasmid and phosphorylation of ERK and Akt was investigated by the western blotting assays. The data represents 5 independent experiments. (C) Quantification data of the panel C showed the significant increase in the phosphorylation of ERK and Akt among the GPCRs overexpressed N2A cells compared with control (mock) group. The data represent mean ± S.E.M. (Student’s t-test, n = 5, *P*<0.05* and 0.01**)

To prove that the identified orphan GPCRs were the mediator of Pls-signaling, we cloned four mouse GPCRs into the CMV promoter containing flag tagged expression vector. Since we failed to clone the cDNA of *GPR27* in the present study using the mouse embryonic cDNA, we used only the four GPCRs for the overexpression study. We overexpressed the cloned expression vectors into the Hek293-T cells cultured with 2% FBS containing DMEM medium and confirmed the overexpressed protein in the cells by western blotting assays using the Flag antibody. We have used low concentration of FBS aimed to see the changes in the phosphorylation of ERK and Akt by the overexpression with the fear that high FBS could mask the effect of these overexpressed proteins. Interestingly, compared with the control vector (empty plasmid) transfected cells, all these protein overexpression enhanced the phosphorylation of ERK and Akt (Fig 3A), suggesting that these proteins had signal enhancing effects in the non-neuronal cells. Similar to that of the Hek293T cells, we then overexpressed the GPCRs in the neuronal cells and found that all of these GPCRs significantly enhanced phosphorylation of ERK and Akt (Fig 3B and 3C). These data show that GPR1, GPR19, GPR21 and GPR61 have the ability to enhance the cellular signaling in various types of cells when they are overexpressed. Since astrocytes had lower expression of these GPCRs (Fig 1), it was possible to explain why extracellular addition of Pls failed to induce p-ERK and p-Akt in these cells, which was questioned in our previous study [7]. These findings suggest that high expression of these GPCRs in the neuronal cells may be a cause of the neuronal cells sensitivity to the Pls.

### The GPCRs accelerate Pls-mediated phosphorylation of ERK and Akt in the neuronal cells

**Fig 4:**
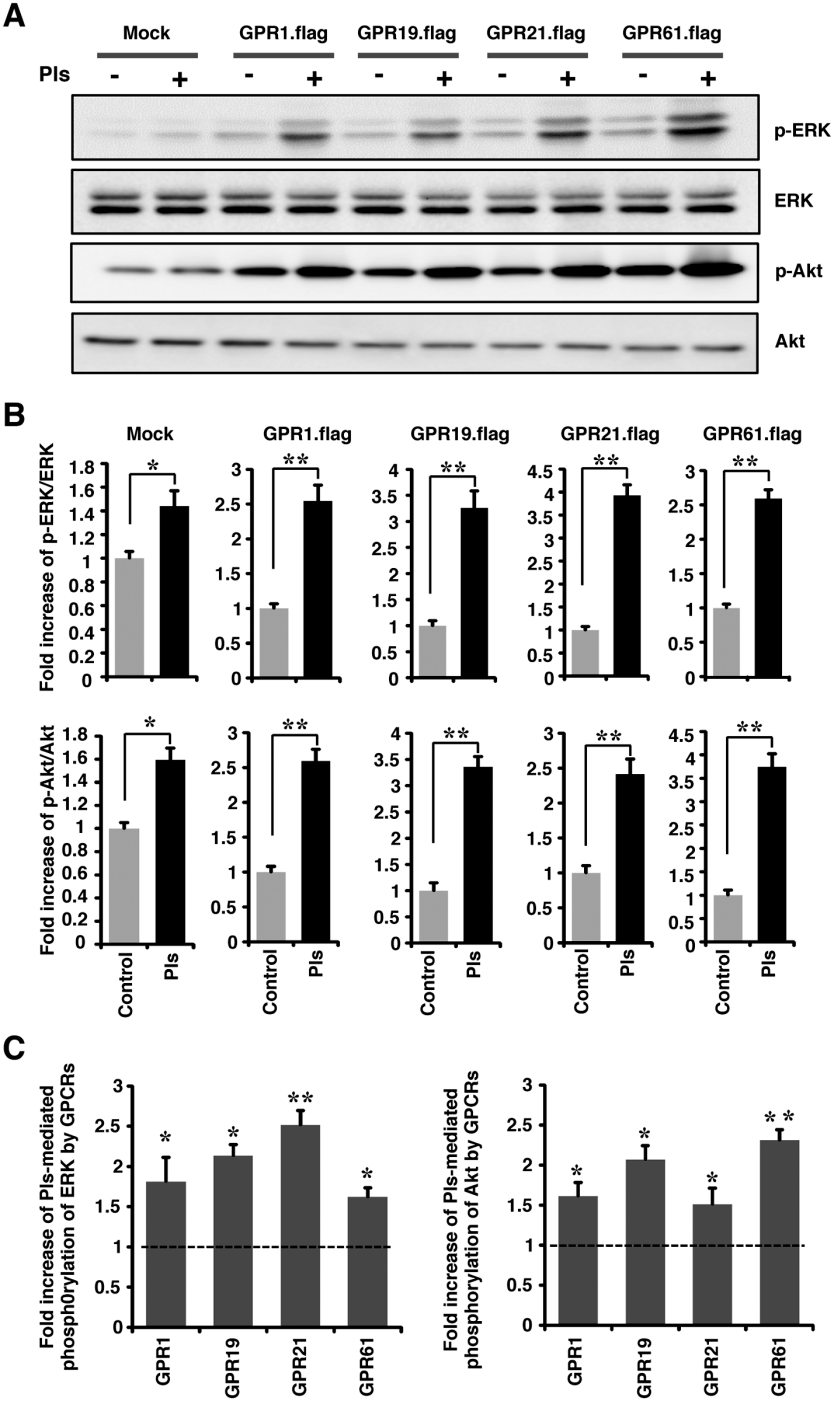
The GPCRs enhances Pls-mediated signaling in the cells. (A) N2A cells cultured with low serum (2% FBS) were transfected by the control and GPCRs plasmids for 48 hours followed by the treatments with Pls (500 ng/ml) for 20 minutes. Cell extracts were subjected to western blotting assays. The data represents five independent experiments (n = 3). (B) Quantification data of panel A showed the significant increase in the phosphorylation of ERK and Akt proteins among the GPCRs (*GPR1*, *GPR19*, *GPR21* and *GPR61*) transfected and the mock transfected cells by the Pls treatments. The data represent mean ± S.E.M (Student’s t-test, n = 5, *, *P*<0.05 and **, *P*<0.01). (C) The fold induction in the level of phosphorylated ERK and Akt compared with the mock transfected cells (considered as one unit) was counted in the GPCRs overexpressed cells. Statistical analysis showed a significant increase in the Pls-mediated induction of ERK and Akt phosphorylation by the GPCRs overexpression in the neuronal cells shown in panel A (Bonferroni’s test, n = 5, *, *P*<0.05 and **, *P*<0.01).

Though the overexpression of the GPCRs itself was enough to induce the phosphorylation of ERK and Akt, it was unknown whether these overexpression facilitated the Pls-induced signaling. To examine this issue, we treated the GPCRs overexpressed neuronal cells with Pls and checked the signaling by western blotting assays. We found that the addition of Pls induced phosphorylation of ERK and Akt in the GPCRs overexpressed cells compared with the Mock (pCDNA3.flag) transfected group (Fig 4A and 4B). Most interestingly the fold increase in the phosphorylation of Akt and ERK by the overexpression of GPCRs was significantly higher compared with the mock group (Fig 4C). These data clearly suggested that the Pls-mediated activation of cellular signaling was enhanced by the overexpression of the GPCRs in the cells.

### Endogenously produced Pls can enhance the GPCRs-mediated signaling

**Fig 5:**
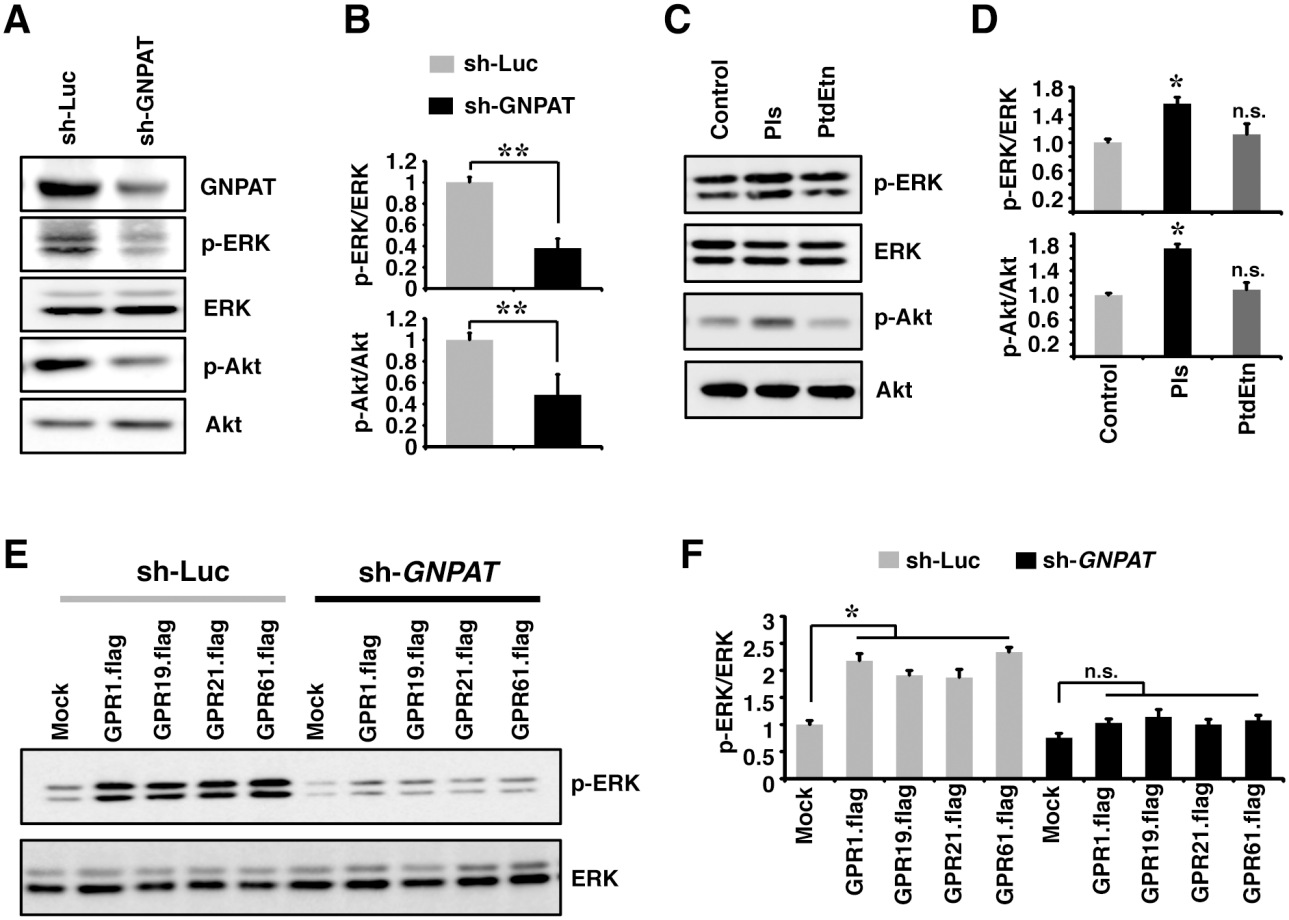
The Pls specific cellular signaling mediated by the GPCRs. (A) Western blotting assays showed the reduction of p-ERK and p-Akt expression in the N2A cells where the Pls-synthesizing enzyme GNPAT was knocked down by sh-*GNPAT* lentivirus. The data represent three independent experiments. (B) The quantification data of the panel A showed the significant reduction of phosphorylated ERK and Akt proteins in the *GNPAT* knockdown cells. The data represent mean ± S.E.M (Student’s t-test, n = 3, **, *P* < 0.01). (C) Serum deprived N2A cells were treated with Pls (500 ng/ml) and PtdEtn (phosphatidylethanolamine, 500 ng/ml) for 20 minutes. Cells extracts were then subjected to western blotting assays to detect phosphorylation status of ERK and Akt proteins. The data represent three independent experiments. (D) Quantification data of panel C showed that PtdEtn treatments did not significantly increase the phosphorylated ERK and Akt as compared with the Pls treatments group. The data represent mean ± S.E.M (Bonferroni’s test; n = 3, *, *P* < 0.05). (E) N2A cells were infected by sh-*GNPAT* and the control sh-Luc lentivirus particles for 24 hours flowed by the overexpression of the GPCRs expressing plasmids for 48 hours. Cell extracts were then subjected to western blotting assays. The data represents three independent experiments (n = 3). (F) Quantification data of the panel E showed that the GPCRs-mediated increase in phosphorylation of ERK in the control lentivirus group (sh-Luc) was abolished in the sh-*GNPAT* groups. The data represent mean ± S.E.M (Dunnett`s test; n = 3, *, *P* < 0.05).

To rule out the possibility that cellular derived Pls might have the ability to regulate the signaling via the GPCR proteins, we first knocked down endogenous *GNPAT* (glyceronephosphate O-acyltransferase, a rate limiting Pls synthesizing enzyme) gene by sh-*GNPAT* lentiviral particles. Interestingly, the reduction of the endogenous *GNPAT* expression by sh-RNA in the neuronal cells was associated with the significant reduction of phosphorylated ERK and Akt proteins (Fig 5A and 5B). Mass spectroscopic assays showed that the knockdown of *GNPAT* in the N2A cells resulted in a significant reduction of ethanolamine Pls (PlsEtn), vinyl ether bonded type of glycerophospholipids, without affecting ester-type phospholipids (e.g., phosphatidylethanolamine, PtdEtn) in the experimental condition of panel A in Fig 5 (data not shown). To make it clear whether the cellular signaling was specific to the Pls but not to ester-type of phospholipids, we treated the serum deprived N2A cells by the PtdEtn and Pls. Western blotting data showed that the PtdEtn treatments failed to induce ERK and Akt signaling compared with that of the Pls treatments (Fig 5C and 5D), which suggest that among the similar class of the phospholipids Pls had the specific effect to induce ERK and Akt signaling. To prove that the orphan GPCRs could transduce the Pls-mediated signaling, we overexpressed the GPCRs in the Pls-reduced N2A cells, where endogenous Pls synthesis was disrupted by sh-*GNPAT* lentiviral particles, and found a significant reduction of GPCRs-mediated increase in ERK signaling (Fig 5E and 5F). These cumulative data suggest that the endogenous Pls may induce the orphan GPCRs-mediated signaling in neuronal cells.

## Discussion

In this study, we report for the first time that neuronal specific GPCRs can transduce Pls-mediated signaling in cells. Because of the increased expression of the GPCRs in neuronal cells, our results explained a possible mechanism for neuronal cell induction of ERK and Akt signaling by Pls which was not explained in our previous study [7]. Our current data also show that the overexpression of these neuronal specific GPCRs can enhance cellular signaling in the non-neuronal cell line Hek293-T. It is, therefore, suggested that the low endogenous expression of GPCRs in primary astrocytes could explain their insensitivity to Pls treatments, which was investigated in our previous report [7]. Results obtained in the current study provide sufficient evidence to show that Pls-induced cellular signaling is accelerated by GPCR proteins and question whether endogenous cellular Pls are necessary for the signaling activity of these GPCRs in neuronal cells. Since it is well understood that intracellular Pls produced in the endoplasmic reticulum can be transported out of the cells [1], it is possible that Pls secreted from cells can activate GPCRs on neuronal cell membranes to maintain ERK and Akt signaling. Interestingly, our present findings show that reduction of Pls in neuronal cells significantly reduced the activation of ERK signaling by the overexpression of GPCR protein(s). It is possible that cell-derived Pls in the brain could maintain the cellular signaling among neuronal cells by GPCR protein(s). Further study will be carried out to assess the possible biological and functional contribution of Pls (e.g., neuronal cell survival, memory maintenance, etc.) mediated by each of these GPCRs in neuronal cells.

It is well known that GPCR proteins can form functionally active homomers and heteromers with different GPCRs or even tyrosine kinase receptors to induce cellular signaling [11,19,20]. These multi-protein complexes could induce kinase activity resulting in the phosphorylation of Akt and ERK. It is therefore possible that these GPCRs may work independently of Pls in the neuronal cells to transduce cellular signaling. However, our results showed that the extracellular addition of Pls enhanced the signaling of these GPCRs, suggesting that Pls might have a role in modulating the possible homodimers and even heterodimers. The findings of our current research show that the single knockdown of each of the GPCRs (*GPR1*, *GPR19*, *GPR21*, *GPR27* and *GPR61*) can effectively cancel the Pls-mediated signaling, suggesting that each of the GPCRs may form heterodimers with other GPCRs to transduce the Pls signaling. Our current data, showing that other GPCRs do not compensate each other (Fig 2), suggest that each of these GPCRs (*GPR1*, *GPR19*, *GPR21*, *GPR2* and *GPR61*) may play an important role in activating the remaining GPCRs by Pls, possibly by forming active heteromers. Further studies are planned to assess whether each of these GPCRs can form heteromers with the remaining GPCRs to transduce Pls-signaling in neuronal cells. In the present study, we have confirmed that the addition of Pls can accelerate the cellular signaling in the GPR1, GPR19, GPR21, and GPR61 overexpressed neuronal cells (Fig 4). However, it is still unknown whether the Pls have any signal enhancing effect to the other neuronal specific GPCRs such as GPR27, GPR62, GPR135, GPR142, GPR150 and GPR162. Further study will be carried out to see if the Pls have any influence on those GPCRs when they are overexpressed in the cells. However, by the knockdown study, it seems that those GPCRs except the GPR27 may not be crucial for the signal transduction by Pls. We observed that relatively weak knockdown of *GPR1* gave a similar effect to that of strong knockdown of *GPR19* and *GPR61* in cancelling the Pls-mediated ERK signal, suggesting that these GPCRs might have different sensitivity to Pls. Another possibility is that Pls can activate cellular signaling via lipid rafts of cellular membranes, which usually anchor many functional membrane proteins including GPCRs. Interestingly, Pls were found to be enriched in lipid rafts [4]. It has also been reported that many GPCRs are located in the lipid raft compartments of cellular membranes which are enriched in cholesterol, sphingomyelin, caveolin, flotillin and other components [9,10], suggesting a possibility that the Pls in lipid rafts may have a role in signal transduction through GPCRs. In addition to the lipid raft hypothesis, it is also possible that Pls could act as ligands to activate GPCRs because some lipids are reported to activate GPCRs as ligands to induce cellular signaling [21]. Since the addition of Pls to the culture medium activated ERK and Akt signaling in neuronal cells within a short time in our current study, it was reasonable to suggest that Pls might act as ligands to activate these GPCRs. If the Pls act as ligands, similar to the lyso-type lipids, PFA [13, 14], Pls may bind directly with GPCRs to activate them. A study of the direct interaction between GPCRs and Pls will be carried out to address this process. Thus, we intend to study these two possible functional consequences in a future study, using both ligands and lipid components, to clarify the mechanism of Pls activation of GPCRs.

In conclusion, our present study suggests that Pls-signaling is associated with GPCR proteins in neuronal cells which might be important in regulating the cellular signaling to maintain effective neuronal activity in the brain. Future studies focusing on the functional relationship of these GPCRs with Pls in maintaining possible neuronal activities (e.g., neuronal survival, memory consolidation, anti-inflammatory function, etc.), could reveal detailed mechanisms of how Pls function in the nervous system.
